# Fasting Triglycerides in the Upper Normal Range Are Independently Associated with an Increased Risk of Diabetes Mortality in a Large Representative US Population

**DOI:** 10.3390/jcdd11040128

**Published:** 2024-04-21

**Authors:** Yutang Wang

**Affiliations:** Discipline of Life Science, Institute of Innovation, Science and Sustainability, Federation University Australia, Ballarat, VIC 3350, Australia; yutang.wang@federation.edu.au

**Keywords:** triglyceride, diabetes, hypertension, cardiovascular disease, survival

## Abstract

The association between normal-range triglyceride levels and diabetes mortality remains unclear. This cohort study aimed to elucidate this relationship by examining 19,010 US adult participants with fasting serum triglycerides below 150 mg/dL. Cox proportional hazards models were employed to estimate mortality hazard ratios (HRs) and 95% confidence intervals (CIs). Participants were followed up for a mean of 15.3 years, during which 342 diabetes deaths were recorded. A 1 natural log unit increase in triglycerides was associated with a 57% higher risk of diabetes mortality (adjusted HR, 1.57; 95% CI, 1.04–2.38). Comparable results were obtained when triglycerides were analyzed in quartiles. Receiver operating characteristic curve analysis identified an optimal triglyceride cutoff of 94.5 mg/dL for diabetes mortality; individuals with triglyceride levels above this threshold faced a greater risk of diabetes mortality (adjusted HR, 1.43; 95% CI, 1.12–1.83). Further investigation revealed a positive association between normal triglyceride levels and all-cause mortality, though no association was observed between normal triglycerides and mortality from hypertension or cardiovascular disease. In conclusion, elevated triglyceride levels within the normal range were associated with an increased risk of diabetes mortality. Individuals with triglyceride levels of 95 mg/dL or higher may require vigilant monitoring for diabetes and its associated complications.

## 1. Introduction

Diabetes poses a significant global public health concern [1,2], contributing to over one million deaths annually [3]. Consequently, there exists a pressing medical need to identify modifiable risk factors for diabetes development and mortality.

Hypertriglyceridemia, defined as fasting triglycerides ≥ 150 mg/dL [4], frequently coexists with diabetes [5,6]. Individuals with hypertriglyceridemia have an increased risk of diabetes diagnosis [7,8], incidence [9,10,11,12,13], and mortality [8]. As such, the association between diabetes and hypertriglyceridemia is well established. However, the correlation between diabetes and triglycerides within the normal range remains understudied.

To date, only a few studies have examined the relationship between triglycerides and diabetes in individuals with normal triglyceride levels [14,15,16,17]. Three independent studies discovered that higher normal triglycerides are associated with a higher risk of new-onset type 2 diabetes in individuals from Israel [14], China [15], and the Netherlands [16]. Another study found a positive association between normal triglyceride levels and the prevalence of type 2 diabetes in Chinese adults [17].

This has prompted a debate regarding whether the hypertriglyceridemia threshold of 150 mg/dL should be lowered for individuals at a higher risk of diabetes. To further this discourse, additional research is warranted, particularly investigating whether triglycerides in the upper normal range are associated with diabetes mortality.

The present study aimed to bridge this knowledge gap by examining the association between triglycerides and diabetes mortality among 19,010 US adult participants with triglyceride levels within the normal range.

Furthermore, hypertriglyceridemia is associated with hypertension [18] and cardiovascular disease (CVD) [19], as well as CVD mortality [20,21,22] and all-cause mortality [23,24]. Consequently, this study also explored whether triglycerides within the normal range were associated with hypertension mortality, CVD mortality, and all-cause mortality.

## 2. Materials and Methods

### 2.1. Participants

Participants in this cohort study were from the third National Health and Nutrition Examination Survey (NHANES III, 1988–1994) and the subsequent eight cycles of NHANES from 1999 to 2014 [19]. A total of 19,072 adults aged ≥ 20 years who had their fasting serum triglyceride levels within the normal range (<150 mg/dL) attended the examination. Participants with missing fasting plasma glucose (*n* = 43) and those without a follow-up time or with a follow-up time of 0 months (*n* = 19) were excluded. Therefore, the remaining 19,010 participants were included in the final analysis. 

### 2.2. Exposure Variable

The exposure variable of this study was the baseline fasting triglyceride level. To measure serum triglycerides, enzymatic methods were employed. These methods involved a sequence of coupled reactions. Initially, triglycerides underwent hydrolysis to yield glycerol. Subsequently, glycerol underwent oxidation via glycerol oxidase, resulting in the production of dihydroxyacetone phosphate and H_2_O_2_. The generated H_2_O_2_ was then transformed by peroxidase into a phenazone compound, which was quantified by assessing absorbance at 500 nm [25].

### 2.3. Outcome Variables

The outcome variables of this study were various types of mortality. Data pertaining to mortality from diabetes, hypertension, CVD, and all causes were directly retrieved from NHANES-linked mortality files [19]. To evaluate mortality status and the cause of death, the National Center for Health Statistics conducted probabilistic matching to link the NHANES data with death certificate records from the National Death Index (NDI) records [26]. All deaths were classified according to the 9th Revision of the International Classification of Diseases (ICD-9) or the 10th Revision of the International Classification of Diseases (ICD-10) and subsequently recoded using the Underlying Cause of Death 113 (UCOD_113) system [19]. Follow-up time was the duration from the time when the participant was examined at the Mobile Examination Center until either the conclusion of follow-up (31 December 2019) or until death, whichever occurred first.

### 2.4. Covariables

Covariables were described previously [27,28] and included age (continuous), sex (male or female), ethnicity (Hispanic, non-Hispanic white, non-Hispanic black, or other), body mass index (continuous), education (<high school, high school, >high school, or unknown), poverty–income ratio (<130%, 130–349%, ≥350%, or unknown), survey periods (1988–1991, 1991–1994, 1999–2000, 2001–2002, 2003–2004, 2005–2006, 2007–2008, 2009–2010, 2011–2012, or 2013–2014), physical activity (inactive, insufficiently active, or active), alcohol consumption (never, <1 drink per week, 1–6 drinks per week, ≥7 drinks per week, or unknown), smoking status (current smoker, past smoker, or other), systolic blood pressure (continuous), total cholesterol (continuous), high-density lipoprotein (HDL) cholesterol (continuous), and family history of diabetes (yes, no, or unknown).

### 2.5. Statistical Analyses

Data were presented as number and percentage for categorical variables, mean and standard deviation for normally distributed continuous variables, or median and interquartile range for not normally distributed continuous variables to describe the baseline characteristics of the participants [29]. Differences in continuous variables were analyzed using one-way ANOVA (normally distributed) [30] or Kruskal–Wallis one-way ANOVA (not normally distributed) [31], and differences among categorical variables were analyzed using Pearson’s chi-square test [32].

Cox proportional hazards models were used to calculate hazard ratios (HRs) and 95% confidence intervals (CIs) of triglycerides for mortality from hypertension, diabetes, CVD, and all causes [33]. Triglycerides were treated as a continuous variable (natural log-transformed) or categorical variable (in observed quartiles of ≤67, 68–89, 90–113, or ≥114 mg/dL).

Out of 19,010 participants, 604 (3.2%) had missing data, including missing body mass index data (*n* = 196), systolic blood pressure data (*n* = 438), total cholesterol data (*n* = 1), and HDL cholesterol data (*n* = 10). The missing data were imputed via multiple imputation by chained equations [34], with 20 imputed data sets being created [35]. In all the regression analyses, triglycerides, body mass index, systolic blood pressure, total cholesterol, and HDL cholesterol were natural log-transformed to improve data distribution [36].

Sensitivity analyses were conducted when the imputed data were not used, i.e., when excluding the 604 participants with missing data from the analysis or those with a follow-up time of <2 years (*n* = 360).

Receiver operating characteristic curves were constructed and the area under the curve (AUC) was calculated to assess the association of triglycerides with diabetes mortality [37], with the optimal cutoff of triglycerides for diabetes mortality thus determined by the Youden Index [38]. Kaplan–Meier curves were constructed to estimate the survival rates of participants across triglyceride categories, which were compared using the log-rank test [39].

The null hypothesis was rejected for two-sided values of *p* < 0.05. All analyses were performed using SPSS version 27.0 (IBM SPSS Statistics for Windows, Armonk, NY, USA, IBM Corporation) [40].

## 3. Results

### 3.1. General Characteristics

This cohort included 19,010 US adult participants with fasting serum triglycerides within the normal range (<150 mg/dL). The participants had a mean (standard deviation) age of 47 (19) years. Those with higher triglycerides had higher fasting plasma glucose, higher body mass index values, higher systolic blood pressure, higher total cholesterol, and lower HDL cholesterol. In addition, they were older and less physically active and received less education (Table 1).

### 3.2. Association of Triglycerides within the Normal Range with Diabetes Mortality

This cohort was followed up for 291,286 person-years, with a mean follow-up of 15.3 years. During the follow-up, 342 diabetes deaths were recorded. 

**Figure 1:**
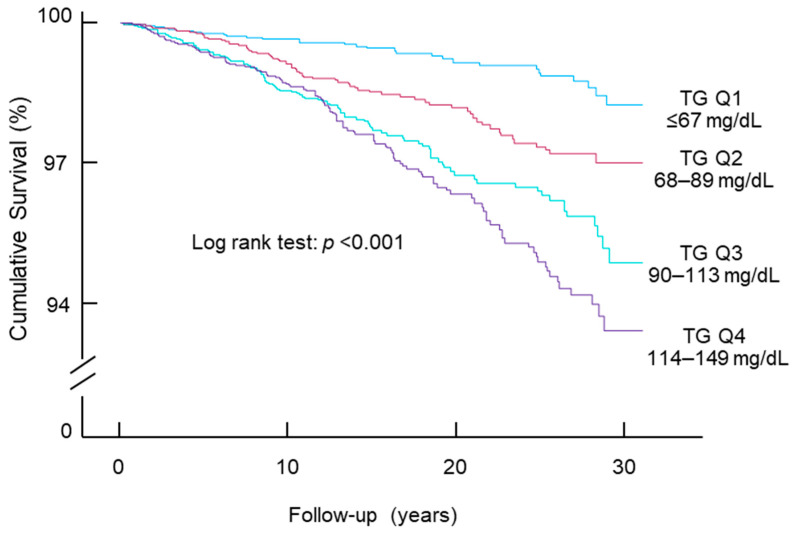
Kaplan–Meier survival curves of diabetes mortality. Participants were stratified according to the quartiles of triglyceride levels. Q, quartile; TG, triglyceride.

A 1 natural log unit increase in triglycerides was associated with a 57% higher risk of diabetes mortality (HR, 1.57; 95% CI, 1.04–2.38; *p* = 0.03; Table 2) after adjustment for all the tested confounding factors. When triglycerides were treated as a categorical variable (i.e., observed quartiles), those with triglycerides in the higher quartiles showed worse survival as analyzed by Kaplan–Meier survival curve analysis (*p* < 0.001, Figure 1). Cox proportional hazards models confirmed these results: participants with triglycerides in the top two quartiles had a higher risk of diabetes mortality compared with those with triglycerides in the bottom quartile after adjustment for all the tested confounding factors (Table 3).

Sensitivity analyses showed that the positive association between triglycerides within the normal range and diabetes mortality remained after excluding those with a follow-up time of less than 2 years (Table 4) or when the imputed data were not used by excluding those 604 participants with missing data from the analysis (Table 5).

### 3.3. The Optimal Cutoff of Triglycerides for Diabetes Mortality

**Figure 2:**
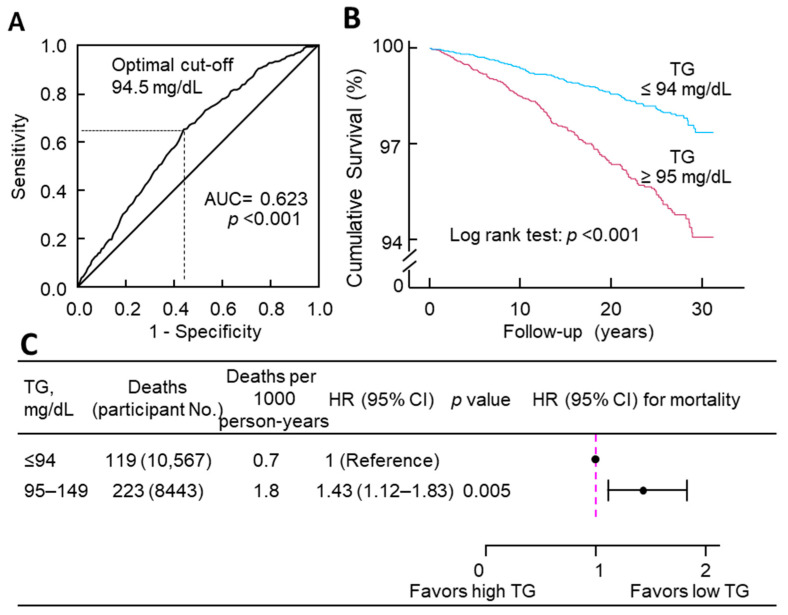
Determination and validation of the cutoff of triglycerides for diabetes mortality. (**A**) ROC curve of triglycerides to classify diabetes mortality. The optimal cutoff was 94.5 mg/dL, with a sensitivity of 65%, specificity of 56%, and an area under the curve (AUC) of 0.623. (**B**) Kaplan–Meier survival curves. (**C**) Diabetes mortality risk associated with triglyceride categories. The analysis was adjusted for age, sex, ethnicity, body mass index, education, poverty–income ratio, survey period, physical activity, alcohol consumption, smoking status, systolic blood pressure, total cholesterol, HDL cholesterol, and family history of diabetes. CI, confidence interval; HR, hazard ratio; No., number; ROC, receiver operating characteristic; TG, triglyceride.

Receiver operating characteristic curve analysis showed that the optimal cutoff of triglycerides to classify diabetes mortality was 94.5 mg/dL (Figure 2A). Kaplan–Meier survival curves confirmed this classification, and those with triglycerides above the cutoff (i.e., triglycerides ranging between 95 and 149 mg/dL) had significantly worse survival compared with those with triglycerides below the cutoff (*p* < 0.001, Figure 2B). Consistently, after adjusting for all the tested confounders, those with normal triglycerides above the cutoff had a 43% higher risk of diabetes mortality (HR, 1.43; 95% CI, 1.12–1.83; *p* = 0.005; Figure 2C) compared with those with triglycerides below the cutoff.

### 3.4. Association of Triglycerides with Hypertension Mortality, CVD Mortality, and All-Cause Mortality

During the 291,286 person-years of follow-up, 619 hypertension deaths, 1535 CVD deaths, and 4447 all-cause deaths were recorded. 

Normal triglycerides were not associated with hypertension mortality or CVD mortality (Table 6 and Table 7). However, triglycerides in the upper normal range were associated with an increased risk of all-cause mortality (Table 6 and Table 7).

## 4. Discussion

Using a large general cohort of US adults (*n* = 19,010), this study, for the first time, demonstrates that fasting triglycerides in the upper normal range are associated with diabetes mortality. Receiver operating characteristic curve analysis revealed that the optimal cutoff of triglycerides for diabetes mortality was 94.5 mg/dL; individuals surpassing this threshold exhibited a 43% higher multivariable-adjusted risk of diabetes mortality compared to those below it.

The association between hypertriglyceridemia and diabetes is well recognized [9,10,11,12,13,41]; however, the possible causality remains to be determined. Possible causal mechanisms have been proposed, such as elevated triglyceride levels possibly impeding glucose transport [42], inhibiting glucose oxidation [43], and decreasing glycogen synthesis rates [44]. However, one cannot rule out the possibility that diabetes causes a change in triglyceride levels.

So far, only a few studies have explored the association between normal triglycerides and diabetes [14,15,16,17], and they have found that triglycerides in the upper normal range are positively associated with the prevalence [17] and new-onset of diabetes [14,15,16]. The current study expands our knowledge and found that triglycerides in the upper normal range were also associated with diabetes mortality. However, the underlying mechanism is unknown. The applicability of proposed mechanisms connecting hypertriglyceridemia and diabetes to triglycerides within the normal range warrants future investigation.

The current study indicated that the optimal cutoff of triglycerides for diabetes mortality was 94.5 mg/dL. This cutoff is supported by the diabetes mortality data: individuals with triglycerides above the cutoff (i.e., 95–149 mg/dL) had a 43% higher risk of diabetes mortality compared with those below the cutoff (i.e., ≤94 mg/dL). Moreover, the proximity of this cutoff to the triglyceride cutoff of 96.5 mg/dL for type 2 diabetes prevalence in 16,706 Chinese adults [17] adds weight to its significance. While Beshara et al.’s study [14] did not pinpoint the optimal cutoff of triglycerides for new-onset type 2 diabetes, their findings among 3722 Israeli individuals with normal triglycerides indicate that higher normal triglyceride levels (ranging from 100 to 149 mg/dL) were associated with an increased risk of new-onset type 2 diabetes compared to levels below 100 mg/dL, supporting a lower threshold of triglycerides for hypertriglyceridemia. Consequently, the results of this study contribute to the debate surrounding the need to lower the current threshold of hypertriglyceridemia (i.e., 150 mg/dL) for individuals at an increased risk of diabetes.

The association between hypertriglyceridemia and all-cause mortality has been intensively investigated [45]. It has been shown that hypertriglyceridemia is associated with an increased risk of all-cause mortality across various populations worldwide, including those in the US [23], Australia [22], and the Czech Republic [24]. The positive association persists among general hospitalized patients [46] and individuals with specific conditions such as coronary heart disease [47] and diabetes [48]. Moreover, Thomsen et al. showed that lower concentrations of non-fasting plasma triglycerides in individuals with genetic variants known to reduce triglyceride levels were associated with reduced all-cause mortality [49]. Additionally, reducing triglycerides through fenofibrate as an adjunct to statin treatment has been linked to decreased all-cause mortality risk [50]. 

The results of the current study show that triglycerides in the upper normal range are also associated with an increased risk of all-cause mortality, suggesting a potential need to lower the hypertriglyceridemia threshold of 150 mg/dL. While previous investigations have not specifically delved into the association between normal-range triglycerides and all-cause mortality as extensively as the current study, a few reports examining the full spectrum of triglyceride levels have observed such a correlation. For instance, Fang et al. demonstrated that among 7476 US adults, individuals with triglyceride levels ranging from 100 to 149 mg/dL faced an elevated risk of all-cause mortality (HR: 1.12; 95% CI, 1.01–1.12) compared to those with levels below 100 mg/dL [51]. Similarly, Klempfner et al. noted that, in patients with established coronary heart disease, triglyceride levels between 100 and 149 mg/dL were associated with increased all-cause mortality risk (HR: 1.06; 95% CI, 1.01–1.12) compared to levels below 100 mg/dL [47].

Many studies across various countries, including Japan [52], Spain [53], China [54], and the US [55], have consistently shown that hypertriglyceridemia increases the risk of new-onset hypertension in the general population. Several proposed mechanisms may underline this association, such as triglycerides contributing to endothelial dysfunction and increased sympathetic nerve activity [56]. Notably, even within the normal range, triglyceride levels have been positively linked to incident hypertension [55,57].

However, in a novel revelation, the current study indicates that triglycerides within the normal range were not associated with hypertension mortality over a mean follow-up of 15.3 years. This implies that while upper normal-range triglycerides may elevate the risk of new-onset hypertension, they may not exacerbate hypertension to a fatal extent.

In epidemiological studies, hypertriglyceridemia often correlates with increased CVD mortality risk in the general population [20,21,22] and in sub-populations including patients with coronary artery disease [58], cerebrovascular disease [59], or diabetes [19,60,61]. However, conflicting reports exist, with some studies failing to establish such a positive correlation [23,62,63,64]. Nonetheless, a meta-analysis of 19 studies involving 91,285 diabetic patients revealed that triglyceride-lowering therapy was associated with reduced CVD events and mortality [65]. Consequently, hypertriglyceridemia has been recognized as a “risk-enhancing factor” by the American Heart Association and American College of Cardiology [66].

Interestingly, the current study did not find an association between triglycerides within the normal range and CVD mortality. While this suggests that upper normal-range triglycerides may not significantly worsen CVD to the point of mortality, it does not rule out their potential contribution to CVD development. Indeed, research has shown that higher normal triglyceride levels (97–141 mg/dL versus <97 mg/dL) were linked with an increased risk of new-onset ischemic heart disease [67]. Additionally, another study identified 89 mg/dL as the prognostic cutoff value for triglycerides to predict cardiovascular events in a cohort of 14,189 Italian adults [68]. 

The Framingham study established the 150 mg/dL cutoff point for triglyceride levels concerning cardiovascular risk [69], a finding corroborated by subsequent research across various populations [70,71]. The current study’s data do not advocate for altering this cutoff point in the context of cardiovascular disease. However, the findings still indicate the presence of distinct cutoff points of triglycerides for different health conditions, with those for diabetes and overall mortality risk potentially lower than that for cardiovascular risk. Clinical recommendations for defining hypertriglyceridemia perhaps should consider overall mortality risk, not just cardiovascular risk. 

Epidemiological data may offer preliminary insights into the need to reassess the cutoff point. However, conclusive evidence stems from randomized clinical trials (RCTs). Any adjustment to the cutoff value should be predicated on RCTs unequivocally showcasing the superiority of reducing triglycerides to a lower threshold compared to the existing cutoff of 150 mg/dL.

Strengths and limitations: One notable strength of this study is its analysis of triglycerides within the normal range in a large representative cohort of US adults (*n* = 19,010) over a lengthy follow-up period of 15.3 years. Furthermore, the study’s adjustment for various confounders is valuable, as many factors influence the state of human health [72]. This study also has several limitations. First, mortality outcomes were ascertained by linkage to National Death Index (NDI) records through probabilistic matching, which could result in misclassification [8]. However, this matching method has been shown to be highly accurate with an accuracy rate of 98.5% [73]. Second, triglycerides were only measured once, which may result in bias. However, in epidemiological studies, this bias tends to lead to an underestimate rather than an overestimate of risk due to regression dilution [74]. Therefore, the association of triglycerides within the normal range with diabetes mortality might be much stronger if triglyceride levels were measured repeatedly.

## 5. Conclusions

This study found that fasting triglycerides in the upper normal range were associated with an increased risk of diabetes mortality. The optimal cutoff of triglycerides to classify diabetes mortality was 94.5 mg/dL, which is below the current threshold of 150 mg/dL. Individuals with triglyceride levels above the newly determined cutoff (i.e., 94.5 mg/dL) faced a 43% higher risk of diabetes mortality compared to those with levels below the cutoff, underscoring the importance of close monitoring for diabetes and associated complications in such individuals. 

This study exclusively relied on baseline triglyceride levels. Thus, there is merit in conducting a similar study in the future with multiple triglyceride assessments (e.g., annually) over a specified follow-up duration (e.g., five years). Such an approach would afford a more robust investigation into the association between triglycerides and mortality, enhancing the study’s rigor and depth of understanding.

## Figures and Tables

**Table 1:** Baseline characteristics stratified by observed triglyceride quartiles of 19,010 US adult participants with triglycerides within the normal range.

	Quartiles of Triglycerides (Range, mg/dL)				All	*p*
	Q1 (≤67)	Q2 (68–89)	Q3 (90–113)	Q4 (114–149)		
Sample size	4711	4798	4651	4850	19,010	NA
Age, y, mean (SD)	41 (17)	47 (19)	49 (19)	51 (19)	47 (19)	<0.001
Sex (male), *n* (%)	1995 (42)	2178 (45)	2225 (48)	2369 (49)	8767 (46)	<0.001
Triglycerides, mg/dL, median (IQR)	56 (47–62)	79 (73–84)	101 (95–107)	129 (121–139)	89 (68–114)	<0.001
FPG, mg/dL, median (IQR)	92 (87–99)	95 (88–102)	97 (90–105)	99 (92–108)	96 (89–104)	<0.001
BMI, kg/m^2^, median (IQR)	25 (22–28)	26 (23–30)	27 (24–31)	28 (25–32)	26 (23–30)	<0.001
SBP, mm Hg, median (IQR)	115 (106–126)	118 (109–131)	120 (111–133)	123 (112–137)	119 (109–132)	<0.001
TC, mg/dL, median (IQR)	173 (152–197)	186 (164–212)	194 (170–219)	201 (177–229)	188 (164–215)	<0.001
HDL-C, mg/dL, median (IQR)	59 (50–71)	55 (46–65)	52 (44–62)	48 (41–58)	54 (45–64)	<0.001
Ethnicity, *n* (%)						
Non-Hispanic white	1774 (38)	2027 (42)	2071 (45)	2307 (48)	8179 (43)	<0.001
Non-Hispanic black	1733 (37)	1389 (29)	1053 (23)	821 (17)	4996 (26)	
Hispanic	927 (20)	1132 (24)	1271 (27)	1478 (31)	4808 (25)	
Other	277 (6)	250 (5)	256 (6)	244 (5)	1027 (5)	
Education, *n* (%)						
<High school	1133 (24)	1391 (29)	1497 (32)	1662 (34)	5683 (30)	<0.001
High school	1218 (26)	1228 (26)	1177 (25)	1229 (25)	4852 (26)	
>High school	2353 (50)	2153 (45)	1964 (42)	1945 (40)	8415 (44)	
Unknown	7 (0)	26 (1)	13 (0)	14 (0)	60 (0)	
Poverty–income ratio, *n* (%)						
<130%	1281 (27)	1359 (28)	1314 (28)	1370 (28)	5324 (28)	0.10
130–349%	1726 (37)	1745 (36)	1723 (37)	1872 (39)	7066 (37)	
≥350%	1315 (28)	1290 (27)	1218 (26)	1248 (26)	5071 (27)	
Unknown	389 (8)	404 (8)	396 (9)	360 (7)	1549 (8)	
Physical activity, *n* (%)						
Active	1494 (32)	1382 (29)	1220 (26)	1162 (24)	5258 (28)	<0.001
Insufficiently active	1741 (37)	1758 (37)	1724 (37)	1855 (38)	7078 (37)	
Inactive	1475 (31)	1657 (35)	1703 (37)	1831 (38)	6666 (35)	
Unknown	1 (0)	1 (0)	4 (0)	2 (0)	8 (0)	
Alcohol consumption, *n* (%)						
0 drinks/week	722 (15)	798 (17)	815 (18)	905 (19)	3240 (17)	<0.001
<1 drink/week	1092 (23)	1073 (22)	1051 (23)	1100 (23)	4316 (23)	
1–6 drinks/week	1135 (24)	1032 (22)	954 (21)	906 (19)	4027 (21)	
≥7 drinks/week	577 (12)	617 (13)	628 (14)	599 (12)	2421 (13)	
Unknown	1185 (25)	1278 (27)	1203 (26)	1340 (28)	5006 (26)	
Smoking status, *n* (%)						
Current smoker	915 (19)	1121 (23)	1090 (23)	1055 (22)	4181 (22)	<0.001
Past smoker	904 (19)	1053 (22)	1140 (25)	1307 (27)	4404 (23)	
Non-smoker	2890 (61)	2620 (55)	2417 (52)	2485 (51)	10,412 (55)	
Unknown	2 (0)	4 (0)	4 (0)	3 (0)	13 (0)	
Family history of diabetes, *n* (%)						0.35
Yes	1930 (41)	1964 (41)	1968 (42)	2087 (43)	7949 (42)	
No	2685 (57)	2744 (57)	2596 (56)	2671 (55)	10,696 (56)	
Unknown	96 (2)	90 (2)	87 (2)	92 (2)	365 (2)	

Abbreviations: BMI, body mass index; FPG, fasting plasma glucose; HDL-C, high-density lipoprotein cholesterol; IQR, interquartile range; NA, not applicable; Q, quartile; SBP, systolic blood pressure; SD, standard deviation; TC, total cholesterol.

**Table 2:** Association of normal triglycerides (natural log-transformed) with diabetes mortality in 19,010 participants.

Models	HR	95% CI	*p*
Model 1	4.24	2.98–6.03	<0.001
Model 2	2.21	1.54–3.19	<0.001
Model 3	1.70	1.17–2.48	<0.01
Model 4	1.66	1.10–2.51	0.02
Model 5	1.57	1.04–2.38	0.03

CI, confidence interval; HR, hazard ratio. Model 1 was not adjusted; Model 2 was adjusted for age, sex, and ethnicity; Model 3 was adjusted for all the factors in Model 2 plus body mass index, education, poverty–income ratio, and survey period; Model 4 was adjusted for all the factors in Model 3 plus physical activity, alcohol consumption, smoking status, systolic blood pressure, total cholesterol, and HDL cholesterol; and Model 5 was adjusted for all the factors in Model 4 plus family history of diabetes.

**Table 3:** Association of normal triglycerides in observed quartiles with diabetes mortality in 19,010 participants.

Models	Q1	Q2			Q3			Q4		
	HR	HR	95% CI	*p*	HR	95% CI	*p*	HR	95% CI	*p*
Model 1	1	2.18	1.44–3.29	<0.001	3.44	2.33–5.08	<0.001	4.17	2.84–6.11	<0.001
Model 2	1	1.66	1.10–2.52	0.02	2.12	1.43–3.14	<0.001	2.33	1.58–3.44	<0.001
Model 3	1	1.46	0.97–2.22	0.07	1.81	1.22–2.70	<0.01	1.84	1.24–2.73	<0.01
Model 4	1	1.44	0.94–2.20	0.09	1.81	1.20–2.74	0.01	1.80	1.18–2.75	0.01
Model 5	1	1.42	0.93–2.16	0.11	1.77	1.17–2.67	0.01	1.72	1.12–2.63	0.01

CI, confidence interval; HR, hazard ratio; Q, quartile. Model 1 was not adjusted; Model 2 was adjusted for age, sex, and ethnicity; Model 3 was adjusted for all the factors in Model 2 plus body mass index, education, poverty–income ratio, and survey period; Model 4 was adjusted for all the factors in Model 3 plus physical activity, alcohol consumption, smoking status, systolic blood pressure, total cholesterol, and HDL cholesterol; and Model 5 was adjusted for all the factors in Model 4 plus family history of diabetes.

**Table 4:** Sensitivity analyses of the association of normal triglycerides with diabetes mortality in 18,650 participants when participants with a follow-up time of less than 2 years (*n* = 360) were excluded.

Models	HR	95% CI	*p*
Model 1	4.41	3.05–6.37	<0.001
Model 2	2.25	1.54–3.30	<0.001
Model 3	1.71	1.16–2.53	0.01
Model 4	1.63	1.06–2.51	0.03
Model 5	1.55	1.01–2.39	0.046

CI, confidence interval; HR, hazard ratio. Model 1 was not adjusted; Model 2 was adjusted for age, sex, and ethnicity; Model 3 was adjusted for all the factors in Model 2 plus body mass index, education, poverty–income ratio, and survey period; Model 4 was adjusted for all the factors in Model 3 plus physical activity, alcohol consumption, smoking status, systolic blood pressure, total cholesterol, and HDL cholesterol; and Model 5 was adjusted for all the factors in Model 4 plus family history of diabetes.

**Table 5:** Sensitivity analysis of the association of normal triglycerides with diabetes mortality in 18,406 participants when the imputed data (*n* = 604) were excluded.

Models	HR	95% CI	*p*
Model 1	4.41	3.07–6.35	<0.001
Model 2	2.32	1.59–3.39	<0.001
Model 3	1.76	1.19–2.60	<0.01
Model 4	1.64	1.07–2.52	0.02
Model 5	1.56	1.02–2.40	0.04

CI, confidence interval; HR, hazard ratio. Model 1 was not adjusted; Model 2 was adjusted for age, sex, and ethnicity; Model 3 was adjusted for all the factors in Model 2 plus body mass index, education, poverty–income ratio, and survey period; Model 4 was adjusted for all the factors in Model 3 plus physical activity, alcohol consumption, smoking status, systolic blood pressure, total cholesterol, and HDL cholesterol; and Model 5 was adjusted for all the factors in Model 4 plus family history of diabetes.

**Table 6:** Association of normal triglycerides (natural log-transformed) with hypertension mortality, CVD mortality, and all-cause mortality in 19,010 participants.

Models	Hypertension Mortality			CVD Mortality			All-Cause Mortality		
	HR	95% CI	*p*	HR	95% CI	*p*	HR	95% CI	*p*
Model 1	2.54	1.99–3.25	<0.001	2.43	2.08–2.83	<0.001	2.35	2.14–2.57	<0.001
Model 2	1.19	0.92–1.54	0.19	1.08	0.92–1.28	0.34	1.10	1.00–1.21	0.05
Model 3	1.06	0.81–1.38	0.70	1.01	0.85–1.19	0.93	1.10	1.00–1.22	0.05
Model 4	1.07	0.79–1.43	0.68	0.97	0.80–1.17	0.72	1.15	1.03–1.28	0.01
Model 5	1.06	0.79–1.43	0.70	0.96	0.79–1.16	0.66	1.14	1.02–1.28	0.02

CI, confidence interval; CVD, cardiovascular disease; HR, hazard ratio. Model 1 was not adjusted; Model 2 was adjusted for age, sex, and ethnicity; Model 3 was adjusted for all the factors in Model 2 plus body mass index, education, poverty–income ratio, and survey period; Model 4 was adjusted for all the factors in Model 3 plus physical activity, alcohol consumption, smoking status, systolic blood pressure, total cholesterol, and HDL cholesterol; and Model 5 was adjusted for all the factors in Model 4 plus family history of diabetes.

**Table 7:** Association of normal triglycerides in observed quartiles with hypertension mortality, CVD mortality, and all-cause mortality in 19,010 participants.

Models	Q1	Q2			Q3			Q4		
	HR	HR	95% CI	*p*	HR	95% CI	*p*	HR	95% CI	*p*
Hypertension mortality										
Model 1	1	1.65	1.27–2.13	<0.001	2.02	1.57–2.60	<0.001	2.27	1.77–2.91	<0.001
Model 2	1	1.18	0.91–1.53	0.23	1.14	0.89–1.48	0.30	1.16	0.9–1.49	0.25
Model 3	1	1.11	0.85–1.44	0.46	1.06	0.82–1.37	0.68	1.04	0.81–1.35	0.75
Model 4	1	1.11	0.85–1.45	0.46	1.05	0.80–1.38	0.74	1.04	0.79–1.39	0.77
Model 5	1	1.11	0.85–1.45	0.46	1.05	0.80–1.37	0.75	1.04	0.78–1.38	0.79
CVD mortality										
Model 1	1	1.66	1.41–1.96	<0.001	2.06	1.75–2.42	<0.001	2.25	1.92–2.64	<0.001
Model 2	1	1.16	0.98–1.37	0.08	1.13	0.96–1.32	0.16	1.11	0.94–1.30	0.22
Model 3	1	1.14	0.96–1.35	0.13	1.09	0.93–1.29	0.29	1.05	0.89–1.24	0.55
Model 4	1	1.12	0.95–1.33	0.19	1.07	0.90–1.27	0.45	1.01	0.85–1.22	0.88
Model 5	1	1.12	0.94–1.33	0.20	1.07	0.90–1.27	0.46	1.01	0.84–1.21	0.94
All-cause mortality										
Model 1	1	1.59	1.44–1.75	<0.001	1.86	1.70–2.05	<0.001	2.16	1.98–2.37	<0.001
Model 2	1	1.13	1.03–1.25	0.01	1.06	0.97–1.17	0.23	1.10	1.00–1.21	0.04
Model 3	1	1.14	1.04–1.26	0.01	1.07	0.98–1.18	0.15	1.11	1.01–1.22	0.03
Model 4	1	1.16	1.05–1.28	<0.01	1.10	1.00–1.22	0.06	1.15	1.03–1.27	0.01
Model 5	1	1.15	1.05–1.27	<0.01	1.10	0.99–1.21	0.07	1.14	1.03–1.27	0.01

CI, confidence interval; CVD, cardiovascular disease; HR, hazard ratio; Q, quartile. Model 1 was not adjusted; Model 2 was adjusted for age, sex, and ethnicity; Model 3 was adjusted for all the factors in Model 2 plus body mass index, education, poverty–income ratio, and survey period; Model 4 was adjusted for all the factors in Model 3 plus physical activity, alcohol consumption, smoking status, systolic blood pressure, total cholesterol, and HDL cholesterol; and Model 5 was adjusted for all the factors in Model 4 plus family history of diabetes.

## Data Availability

All data in the current analysis are publicly available on the NHANES website (https://www.cdc.gov/nchs/nhanes/index.htm, accessed on 1 March 2023).
